# The hernia is mightier than the aorta

**DOI:** 10.1093/ehjcr/ytaf122

**Published:** 2025-03-07

**Authors:** Hisato Takagi

**Affiliations:** Department of Cardiovascular Surgery, Shizuoka Medical Center, 762-1 Nagasawa, Shimizu-cho, Sunto-gun, Shizuoka 411-8611, Japan

## Case summary

Contrast-enhanced computed tomography (CT) scans in an 83-year-old woman revealed the stomach and duodenum herniating into the mediastinum via the oesophageal hiatus, and oesophageal hiatal hernia was diagnosed. The distal descending thoracic aorta was deviated cranially, dorsally, and dextrally aloof from the thoracic vertebrae by the hernia. The aorta was also remarkably tortuous compressed cranially/dextrally and cranially/dorsally by the hernia on 3D CT aortography.

## Case description

**Figure 1:**
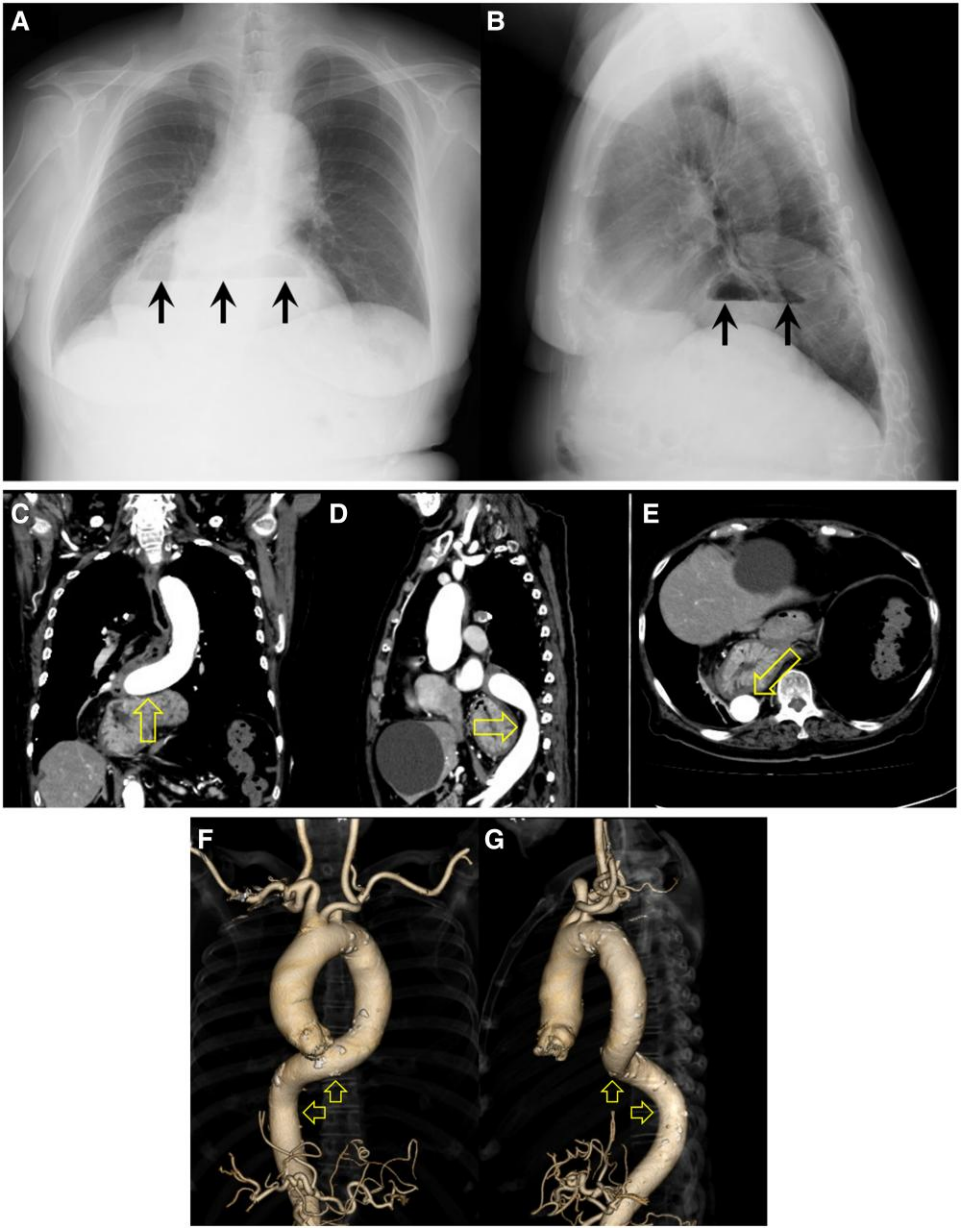
Niveaus (arrows) in the lower mediastinum on chest radiography (*A*, posterior-to-anterior; *B*, right-to-left views). The distal descending thoracic aorta deviated (open arrows) cranially (*C*, coronal plane), dorsally (*D*, sagittal plane), and dextrally (*E*, axial plane) by oesophageal hiatal hernia on contrast-enhanced computed tomography scans. The remarkably tortuous aorta compressed (open arrows) cranially/dextrally (*F,* anterior-to-posterior view) and cranially/dorsally (*G*, left-to-right view) by the hernia on 3D computed tomography aortography.

Niveaus (arrows) in the lower mediastinum on chest radiography (*Figure 1A,* posterior-to-anterior view; *Figure 1B,* right-to-left view) were incidentally identified in an 83-year-old woman with type B acute aortic intramural haematoma. The patient had not complained of gastrointestinal symptoms. Contrast-enhanced computed tomography (CT) scans revealed the stomach and duodenum herniating into the mediastinum via the oesophageal hiatus, and oesophageal hiatal hernia was diagnosed. The distal descending thoracic aorta was deviated (open arrows) cranially (*Figure 1C,* coronal plane), dorsally (*Figure 1D,* sagittal plane), and dextrally aloof from the thoracic vertebrae (*Figure 1E,* axial plane) by the hernia. The aorta was also remarkable tortuous compressed (open arrows) cranially/dextrally (*Figure 1F,* anterior-to-posterior view) and cranially/dorsally (*Figure 1G*, left-to-right view) by the hernia on 3D CT aortography. Elective laparoscopic hernia repair is now scheduled in the chronic phase of the aortic dissection following conservative medical (i.e. antihypertensive and analgesic) treatment for the aortic intramural haematoma.

Intra-abdominal pressure is ∼5–7 mm Hg in critically ill adults, and even >25 mm Hg in Grade-IV abdominal compartment syndrome (viz., intra-abdominal hypertension)^1^ is far lower than aortic pressure (e.g. 120/80 mm Hg in the systole/diastole). Indeed, however, a number of cases with the heart (left ventricular pressure is equal to the aortic pressure) compressed by hiatal or diaphragmatic hernia (leading to complete heart block, heart failure, myocardial infarction, cardiac tamponade, even cardiac arrest,^2^ etc.) have been known. Meanwhile, merely one case (image) with the pushed descending thoracic aorta due to hiatal hernia^3^ has been reported to the best of our knowledge. The tortuous distal descending aorta in the present case is probably owing to compression of the oesophageal hiatal hernia. The hernia may be mightier than the aorta as well as ‘*the pen is mightier than the sword*.’

## Data Availability

The data underlying this article are available in the article.

## Notes

**Consent:** The author confirms that written consent for submission and publication of this case report, including images and associated text, has been obtained from the patient in line with COPE guidance.

**Funding:** None declared.
